# Versican G3 Domain Modulates Breast Cancer Cell Apoptosis: A Mechanism for Breast Cancer Cell Response to Chemotherapy and EGFR Therapy

**DOI:** 10.1371/journal.pone.0026396

**Published:** 2011-11-09

**Authors:** William Weidong Du, Burton B. Yang, Bing L. Yang, Zhaoqun Deng, Ling Fang, Sze Wan Shan, Zina Jeyapalan, Yaou Zhang, Arun Seth, Albert J. Yee

**Affiliations:** 1 Department of Surgery, Sunnybrook Health Sciences Centre and Centre for the Study of Bone Metastasis, Odette Cancer Centre, University of Toronto, Toronto, Canada; 2 Sunnybrook Research Institute, Toronto, Canada; 3 Department of Laboratory Medicine and Pathobiology, University of Toronto, Canada; 4 Division of Life Science, Graduate School at Shenzhen, Tsinghua University, Shenzhen, China; University of Chicago, United States of America

## Abstract

Overexpression of EGFR and versican has been reported in association with breast cancers. Considered oncogenic, these molecules may be attractive therapeutic targets. Possessing anti-apoptotic and drug resistant properties, overexpression of these molecules is accompanied by selective sensitization to the process of apoptosis. In this study, we exogenously expressed a versican G3 construct in breast cancer cell lines and analyzed the effects of G3 on cell viability in fetal bovine serum free conditioned media and evaluated the effects of apoptotic agent C2-ceramide, and chemotherapeutic agents including Docetaxel, Doxorubicin, and Epirubicin. Versican G3 domain enhanced tumor cell resistance to apoptosis when cultured in serum free medium, Doxorubicin, or Epirubicin by up-regulating pERK and GSK-3β (S9P). However, it could be prevented by selective EGFR inhibitor AG 1478 and selective MEK inhibitor PD 98059. Both AG 1478 and PD 98059 enhanced expression of pSAPK/JNK, while selective JNK inhibitor SP 600125 enhanced expression of GSK-3β (S9P). Versican G3 promoted cell apoptosis induced by C2-ceramide or Docetaxel by enhancing expression of pSAPK/JNK and decreasing expression of GSK-3β (S9P), an observation blocked by AG 1478 or SP 6000125. Inhibition of endogenous versican expression by siRNA or reduction of versican G3's expression by linking G3 with 3′UTR prevented G3 modulated cell apoptosis. The dual roles of G3 in modulating breast cancer cell resistance to chemotherapeutic agents may in part explain a potential mechanism for breast cancer cell resistance to chemotherapy and EGFR therapy. The apoptotic effects of chemotherapeutics depend upon the activation and balance of down stream signals in the EGFR pathway. GSK-3β (S9P) appears to function as a key checkpoint in this balance of apoptosis and anti-apoptosis. Investigation and potential consideration of targeting GSK-3β (S9P) merits further study.

## Introduction

Chemotherapeutic drugs exhibit varied selectivity for tumour cells dependent on cell origins and are capable of inducing tumour cell death [1], [2]. Additionally many of the commonly used chemotherapeutic drugs also appear to influence cellular signaling pathways that induce apoptosis in susceptible cancer cells [1], [3]. Apoptosis seems to be one of the major physiologic safeguards against uncontrolled proliferation [4]. Growth and apoptosis are two diametrically opposed biological processes that ensure that multi-cellular organisms can cope with the normal physiologic yet mutagenic environment that generates millions of potential cancer cells every day [5]. With its effects on tumor cell proliferation and migration, versican has been shown to increase the resistance of cancer cells to apoptosis [6]. Our previous research demonstrated that versican appeared to confer cell resistance to apoptosis following treatment with low serum medium or hydrogen peroxide [7], [8]. The combination of selective apoptotic resistance and sensitivity has been reported in overexpression of the V1 versican isoform [7]; the intimate relationship between proliferation and apoptosis cannot be separated and cancer cells often express either hypersensitivity or resistance to apoptosis that is dependent upon tissue conditions.

As a member of the large aggregating chondroitin sulfate proteoglycan family, versican is structurally composed of a N-terminal G1 domain, a glycosaminoglycan (GAG) attachment region, and a C terminus (or G3) selectin-like domain [9], [10]. The G3 domain interacts with different ECM proteins [11] and binds to certain cell surface proteins including epidermal growth factor receptor (EGFR) [12], [13]. Extracellular versican has been observed to be elevated in a variety of human tumors including breast carcinoma [14], [15], [16]. High expression has been observed in the interstitial tissues at the invasive margins of breast carcinoma and appears prognositic being predictive of cancer relapse in patients and negatively impact overall survival rates [17], [18]. The expression of versican G3 domain does not only appear to enhance breast cancer cell proliferation *in vitro* and in the mammary gland, but also promotes tumor cell migration *in vitro* and systemic metastasis in syngenetic orthotopic models *in vivo* [19], [20].

Increased expression of EGFR occurs frequently in human breast cancer and is associated with a poor prognosis [21]. Anti-apoptotic and drug resistant effects have been implicated in EGFR signaling. Some molecules in the pathway, however, may promote cell cycle arrest and enhanced sensitivity to chemotherapeutic drugs [22]. Direct targeting of EGFR is a promising therapeutic strategy for breast cancers with abnormalities in this pathway and may be beneficial in breast cancer patients who cannot tolerate surgery or traditional chemotherapy, or in advanced recalcitrant cases with poor prognoses [23], [24].

There is a desire to improve our understanding of the cellular mechanisms involved in versican G3 mediated tumor growth and invasiveness. Understanding EGFR signaling that influences cell sensitivity to apoptosis as well as effects that are elicited by chemotherapy may help guide our understanding towards identifying other potential target molecules in the pathway from an immunotherapeutic perspective. To investigate the effects of versican G3 domain on breast cancer cell apoptosis, we exogenously expressed versican G3 in mouse mammary tumor cell lines 66c14, 4T07, 4T1 [25], and human breast cancer cell lines MT1, MDA-MB-231, MCF-7, MDA-MB-468. We evaluated the effect of apoptotic agent C2-ceramide as well as chemotherapeutic drugs such as Doxorubicin, Epirubicin, and Docetaxil on cell activity and EGFR downstream signaling.

## Materials and Methods

### Materials and cell cultures

The monoclonal antibodies against ERK2, pERK, CDK2, and Caspase-3 were obtained from Santa Cruz Biotechnology. The polyclonal antibodies against SAPK/JNK and pSAPK/JNK were obtained from Cell Signaling. EGF, selective EGFR inhibitor AG 1478, selective MEK inhibitor PD 98059, selective SAPK/JNK inhibitor SP 600125, hydroxyurea, and the monoclonal antibody against β-actin used in the study were obtained from Sigma. Glycogen synthase kinase-3ß serine-9 phosphorylation (GSK-3β, S9P), and polyclonal antibodies against versican V1 were obtained from Abcam. Horseradish peroxidase-conjugated goat anti-mouse IgG and horseradish peroxidase-conjugated goat anti-rabbit IgG were obtained from Bio-Rad. Immunoblotting was performed using the ECL Western blot detection kit. Cell Proliferation Reagent WST-1 was obtained from Roche Applied Science.

Mouse mammary tumor cell lines 67NR, 66c14, 4T07, 4T1 [26], and human breast cancer cell line MDA-MB-231 were cultured in DMEM media[27], and human breast cancer cell line MT-1 [28], MCF-7 [29], MDA-MB-468 were cultured in RPMI-1640 media [30], which were supplemented with 10% fetal calf serum, penicillin (100 U/ml) and streptomycin (100 µg/ml) and maintained at 37°C in a humidified atmosphere of 5% CO_2_. In selected experiments, cell suspensions were cultured with EGF (20 ng/ml), EGFR inhibitor AG 1478 (2.0 µM), selective MEK inhibitor PD 98059 (50 µM), and selective SAPK/JNK inhibitor SP 600125 (100 nM).

The pcDNA1 - G3 construct and pcDNA1 - G3 fragment lacking the EGF-like motifs (G3ΔEGF) construct were generated by us [31], [32], [33]. Mouse mammary tumor cell lines 66c14, 4T07, 4T1 and human breast cancer cell line MT-1, MDA-MB-231, MCF-7, and MDA-MB-468 cells were transfected with pcDNA1-vecor and G3 constructs. The 66c14 cells were transiently transfected with G3 construct, G3ΔEGF construct, or the control vector. A leading sequence that has been shown to be efficient in product secretion was engineered to both construct by us previously [32], [34], [35].

### Cell viability assays

G3 and vector-transfected 66c14 cells (2×10^5^) were cultured in 10% FBS/DMEM medium in culture dishes and maintained at 37°C for 12 hours. After cell attachment, we changed the medium to serum free DMEM medium or 10% FBS/DMEM medium which contained different concentrations of chemotherapeutic compounds. Cells were harvested daily and cell number was analyzed by Coulter Counter. Cell survival assays were also performed with colorimetric proliferation assays (Cell Proliferation Reagent WST-1). Versican G3 and control vector transfected breast cancer cells (1×10^4^ cells/well) were inoculated and cultured in 10% FBS/DMEM medium in 96 well culture dishes for 12 hours. After cell attachment, we changed the medium into serum free DMEM medium or 10% FBS/DMEM medium containing different concentrations of chemotherapeutic agents, and then cultured cells with 10 µl WST-1 reagent for 4 hours. The absorbance of the samples against a background blank control was measured by a microplate reader.

### Western blot analysis

Protein samples were subjected to sodium dodecyl sulfate-polyacrylamide gel electrophoresis (SDS-PAGE) on separating gel containing 7–10% acrylamide. Separated proteins were transblotted onto a nitrocellulose membrane in 1×Tris/glycine buffer containing 20% methanol at 60 V for 2 h in a cold room. The membrane was blocked in TBST (10 mM Tris-Cl, pH 8.0, 150 mM NaCl, 0.05% Tween 20) containing 5% non-fat dry milk powder (TBSTM) for 1 hour at room temperature, and then incubated with primary antibodies at 4°C overnight. The membranes were washed with TBST (3×30 minutes) and then incubated with appropriate horseradish peroxidase-conjugated secondary antibodies in TBSTM for 1 hour. After washing as described above, the bound antibodies were visualized with an ECL detection kit as described previously [36], [37].

### Cell cycle analysis

The expression of cell cycle-related proteins was analyzed by immunoblotting probed with appropriate antibodies as described above. G3- and vector-transfected 66c14 cell lines were cultured in 10% FBS/DMEM media at 37°C, 5% CO_2_ with or without EGFR inhibitor AG 1478 (0.2, 2.0, and 5.0 µM), and selective MEK inhibitor PD 98059 (20, 50, and 100 µM). The cells were washed and resuspended in cold PBS and incubated in ice-cold 70% ethanol for 3 hours. The cells were then centrifuged at 1,500 rpm for 10 minutes and resuspended in propidium iodide (PI) master mix (40 mg/ml PI and 100 mg/ml RNase in PBS) at a density of 5×10^5^/ml and incubated at 37°C for 30 minutes before analysis by flow cytometry.

### Annexin V assays

An Annexin V-FITC apoptosis detection kit (Biovision Inc, Mountain View, CA, USA) was used to detect apoptotic activity. Cells (1×10^6^) were collected and resuspended in binding buffer, and Annexin V-FITC and propidium iodide were added to each sample and incubated in the dark for 5 minutes. Annexin V-FITC binding was determined by flow cytometry (Ex = 488 nm; Em = 530 nm) using FITC signal detector (FL1) and propidium staining by the phycoerythrin emission signal detector (FL2).

### RT - PCR

2×10^6^ cells were harvested, and total RNA was extracted with the Qiagen RNeasy mini kit. Two micrograms of total RNA were used to synthesize cDNA, a portion of which (equal to 0.2 µg RNA) was used in a PCR with two appropriate primers. PCR products were analyzed on agarose gel and detected using ethidium bromide staining as previously described [38], [39].

## Results

### Versican G3 domain enhanced tumor cell survival in serum free medium by up-regulating pERK and GSK-3β (S9P)

**Figure 1:**
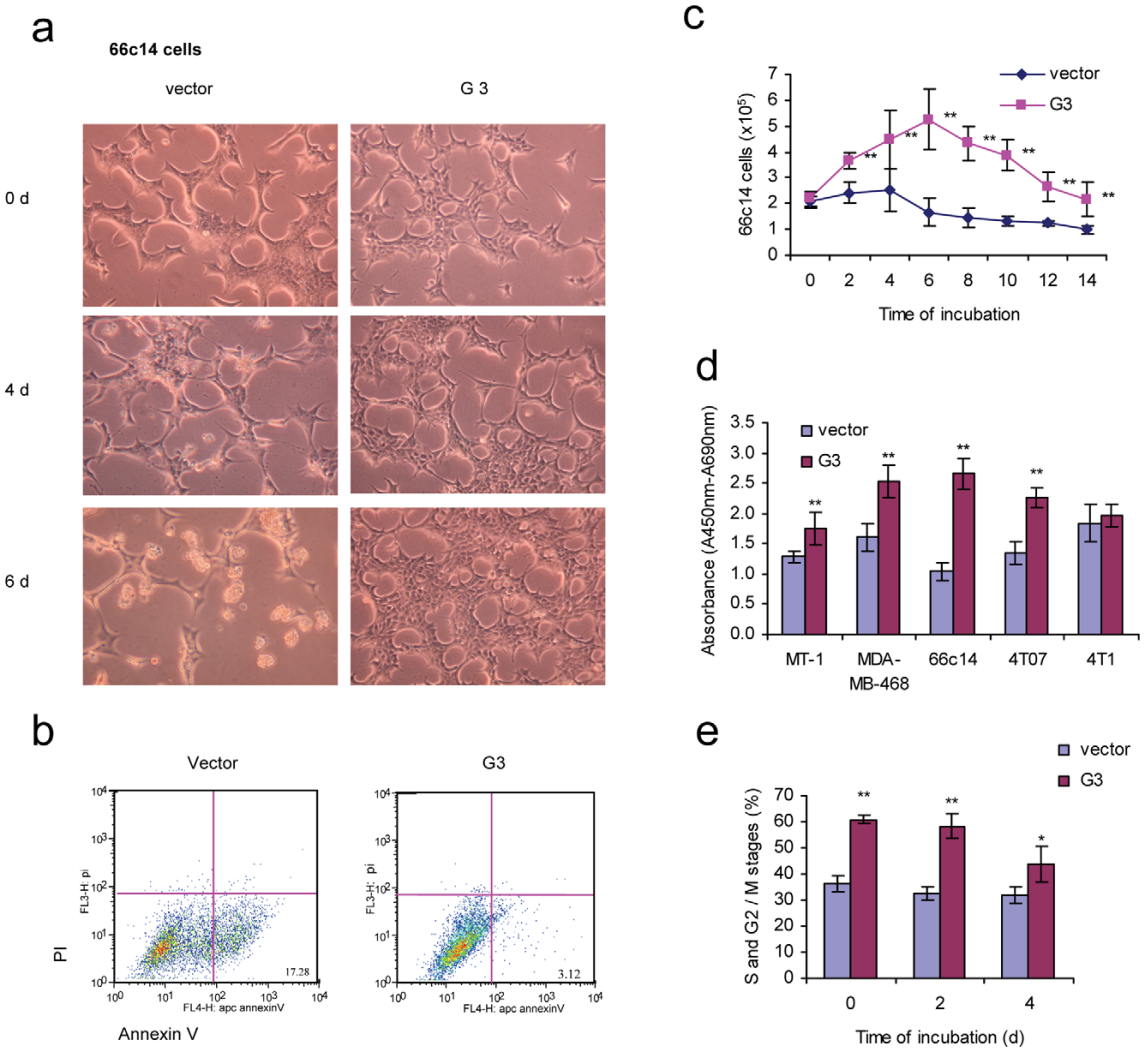
Versican G3 domain enhanced tumor cell survival in serum free medium. a) G3-transfected and vector-transfected 66c14 (2×10^5^) were cultured in 10% FBS/DMEM medium in culture dishes for 12 hours. After cell attachment, we changed the medium to serum free DMEM and cultured cells for 6 days. Cell viability was analyzed by light microscopy. b) After culturing in serum free medium for 2 days, cells were analyzed with Annexin V and propidium iodide staining using flow cytometry. Annexin V and propidium iodide assays confirmed that cell death was apoptosis. c) G3- and vector-transfected 66c14 (2×10^5^) were cultured in 10% FBS/DMEM medium in culture dishes for 12 hours. After cell attachment, we changed the medium into serum free DMEM medium and cultured them for 14 days. Cells were harvested and counted under light microscopy every 2 days. Experimental results are compared with vector control group, *n* = 6, * *p*<0.05, **p<0.01, analyzed with *t*-test. d) 1×10^4^ G3- and vector-transfected human breast cancer cells MT-1 and MDA-MB-468, mouse mammary tumor cells 66c14, 4T07, and 4T1 were inoculated and cultured in 10% FBS/DMEM medium in 96 well culture dishes for 12 hours. After cell attachment, we changed the medium to serum free DMEM and cultured them for 8 days. WST-1 Cell Survival Assays were used to test cell viability. Compared with vector control group, *n* = 6, * *p*<0.05, **p<0.01, analyzed with *t*-test. e) Cell cycles were analyzed by flow cytometer. Compared with vector control group, *n* = 4, * *p*<0.05, **p<0.01, analyzed with *t*-test.

**Figure 2:**
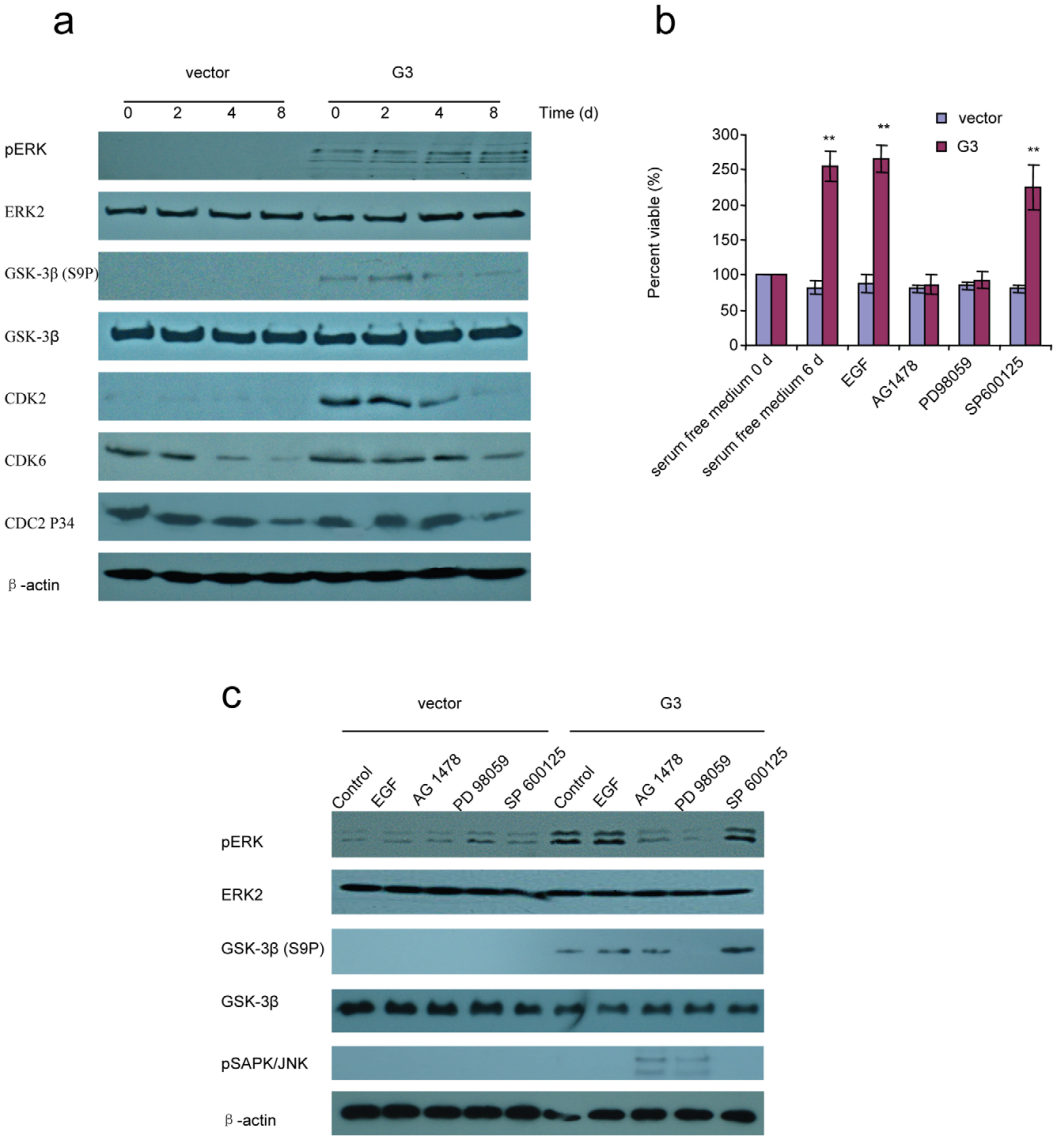
Versican G3 domain enhanced tumor cell survival in serum free medium by up-regulating pERK. a) G3- and vector-transfected 66c14 (2×10^5^) 2×10^5^ were cultured in 10% FBS/DMEM medium in culture dishes for 12 hours. After cell attachment, we changed the medium to serum free DMEM and cultured them for 6 days. Cell lysates were prepared and subjected to immunoblotting with antibodies to ERK2, pERK, GSK-3β (S9P), GSK-3β, CDK2, CDK6, CDC2P34, and β-actin. b) G3-transfected and vector-transfected 66c14 cells (1×10^4^) were inoculated and cultured in 10% FBS/DMEM medium in 96 well culture dishes for 12 hours. After cell attachment, we changed the medium to serum free DMEM, with EGF (20 ng/ml), selective EGFR inhibitor AG 1478 (2.0 µM), selective MEK (ERK kinase) inhibitor PD 98059 (50 µM), or selective JNK inhibitor SP 600125 (100 nM) cultured for 6 days with medium changed every 2 days. WST-1 Cell Survival Assays were used to analyze cell viability. Compared with vector control group, *n* = 6, * *p*<0.05, **p<0.01, analyzed with t-test. c) After culture in serum free medium for 6 days, cell lysates were prepared and subjected to immunoblotting with antibodies to ERK2, pERK, GSK-3β (S9P), GSK-3β, and β-actin.

A greater viability in low serum and serum-free conditions in the presence of versican G3 was observed in human breast cancer cells [19]. To investigate the expression of versican G3 domain on breast cancer cell survival, G3-transfected or vector-transfected 66c14 cells were cultured in serum free DMEM medium. G3 transfected cells grew faster than vector cells in the initial 4 days. After 4 days, a great number of vector cells floated in the medium, while the G3 transfected cells appeared well attached (Fig. 1a). Annexin V assays confirmed that cell death occurred through apoptosis (Fig. 1b). G3-transfected 66c14 cells showed a greater viability during 14 days of culture in serum free medium (Fig. 1c). Versican G3 domain enhanced mouse breast cancer cell line 66c14, 4T07 and human breast cancer cell line MT1 and MDA-MB-468 survival in serum free medium (Fig. 1d). However expression of G3 in 4T1 cell line, which is demonstrated to have high levels of endogeneous versican [20], didn't change the cell proliferation significantly. Flow cytometer confirmed that the percentage of cells in S, G2 and M stages were much higher in G3- transfected cells than in vector cells (Fig. 1e). Immunoblotting indicated that versican G3 enhanced cell survival in serum free medium by increasing expression of pERK, GSK-3β (S9P) and CDK2 (Fig. 2a). Versican G3 enhanced cell survival could be prevented by selective EGFR inhibitor AG 1478 and selective MEK (ERK kinase) inhibitor PD 98059 (Fig. 2b). Immunoblotting showed that both AG 1478 and PD 98059 enhanced expression of pSAPK/JNK in G3 expressing cells, and partly prevented G3 enhanced expression of pERK. Whereas only PD 98059 blocked G3 enhanced expression of GSK-3β (S9P) (Fig. 2c). Selective JNK inhibitor SP 600125 enhanced expression of GSK-3β (S9P).

### Versican G3 enhanced breast cancer cell apoptosis induced by C2-ceramide through expression of pSAPK/JNK and caspase-3

**Figure 3:**
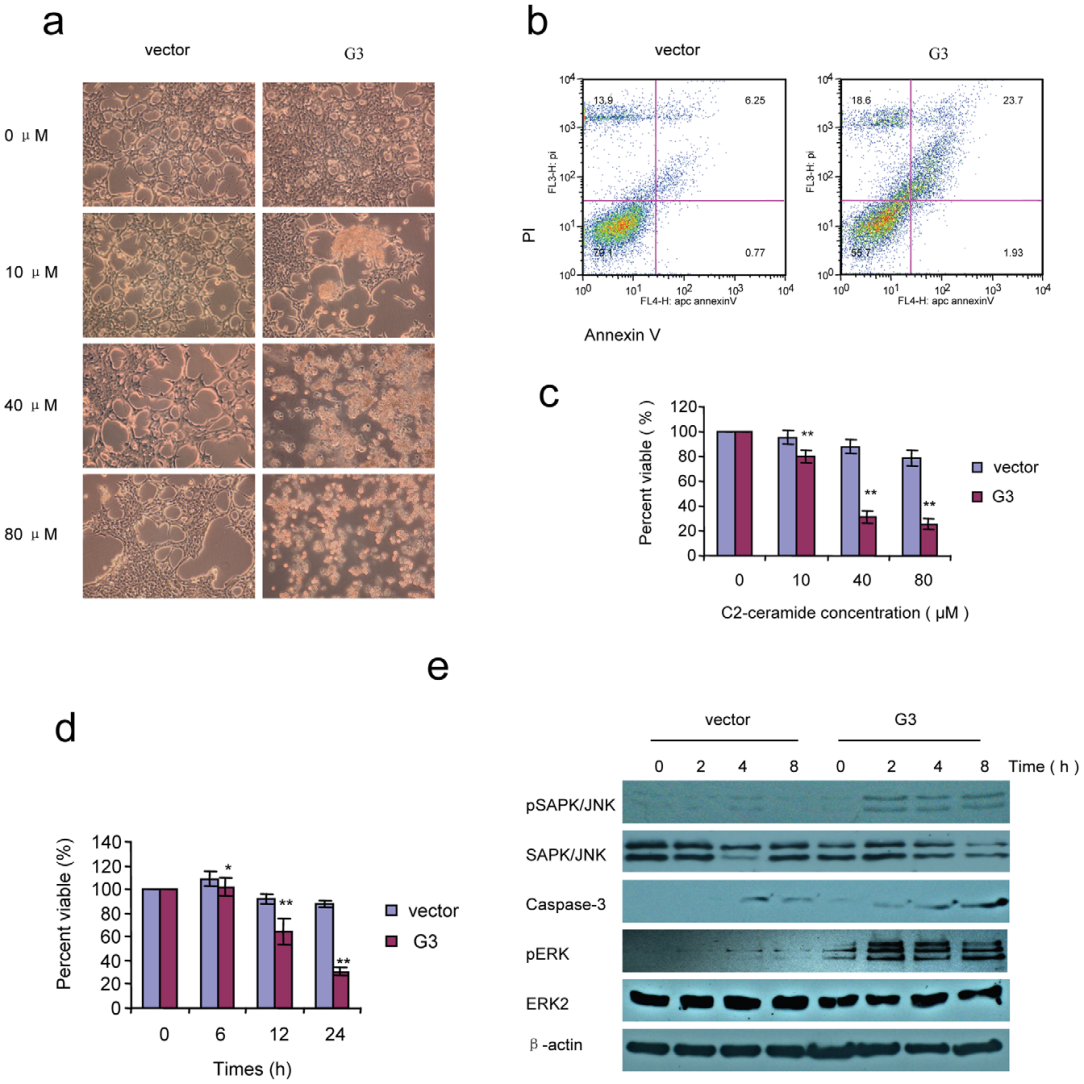
Versican G3 enhanced cell apoptosis induced by C2-ceramide by expressing pSAPK/JNK and caspase-3. a) G3- and vector-transfected 66c14 cells (2×10^5^) were inoculated in 6 well culture dishes. After cultured for 12 hours, all samples were treated with 0, 10, 40, or 80 µM C2-ceramide for 24 hours. Analysis by light microscopy revealed that treatment with a dose of 40 or 80 µM C2-ceramide induced significant cell death in G3-transfected cells. b) After culture in 40 µM C2-ceramide serum free medium for 4 hours, cells were analyzed with Annexin V and propidium iodide staining using flow cytometry. Annexin V and propidium iodide assays confirmed that cell death occurred through apoptosis. c) G3-transfected and vector-transfected 66c14 cells (1×10^4^) were inoculated and cultured in 10% FBS/DMEM medium in 96 well culture dishes for 12 hours. After cell attachment, all samples were treated with 0, 10, 40, or 80 µM C2-ceramide for 24 hours. Lower cell viability was observed for the G3 experimental group as compared with the control group. Compared with vector control group, *n* = 6, * *p*<0.05, **p<0.01, analyzed with *t*-test. d) Cells were also treated with 40 µM C2-ceramide for 6, 12, 24 hours. WST-1 assays were performed. Compared with vector control group, *n* = 6, * *p*<0.05, **p<0.01, analyzed with *t*-test. e) Cells were also treated with 40 µM C2-ceramide for 6 hours, harvested and subjected to immunoblotting with antibodies to pSAPK/JNK, SAPK/JNK, ERK2, pERK, Caspase-3, and β-actin.

**Figure 4:**
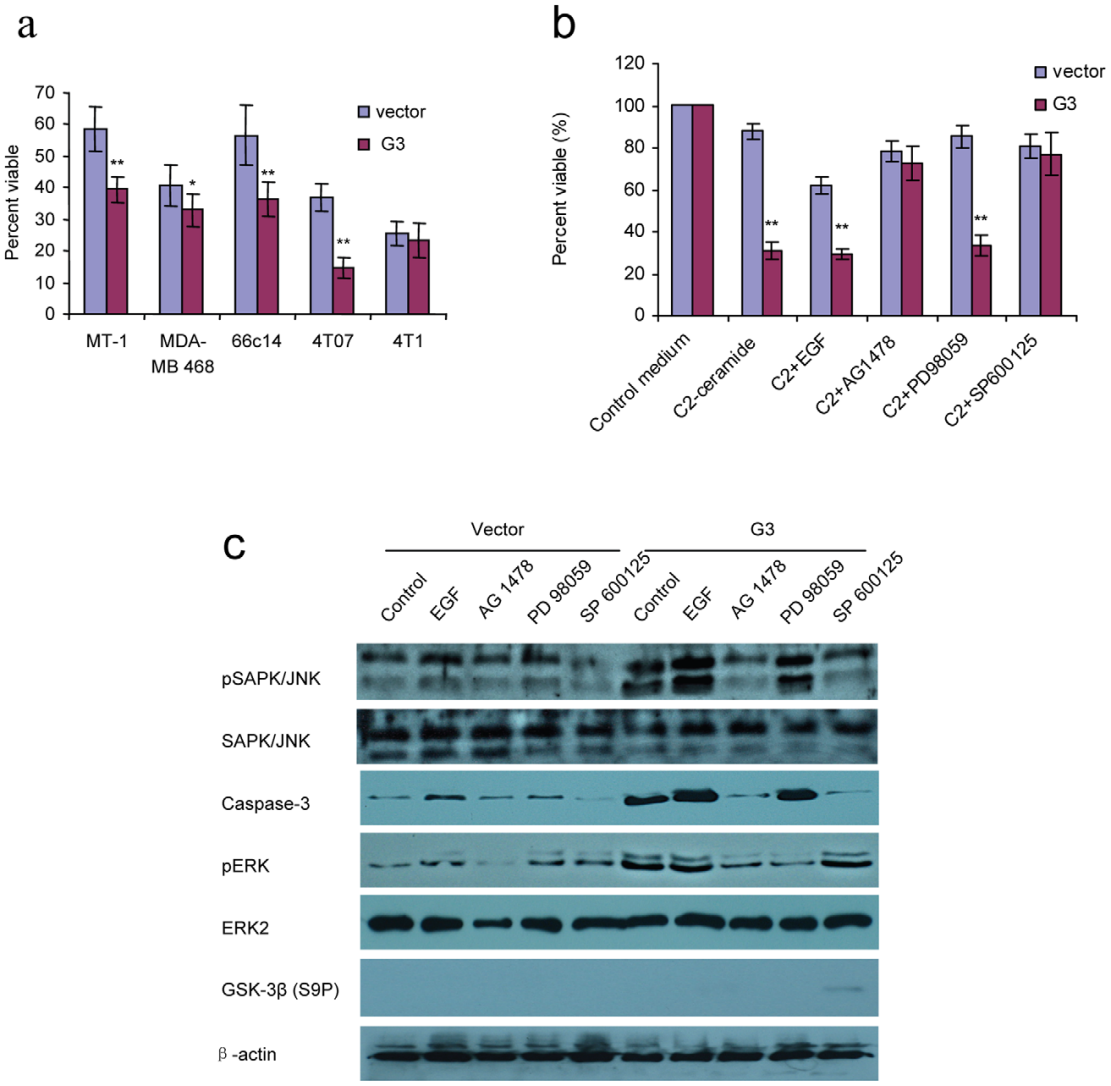
Versican G3 domain enhanced tumor cell apoptosis induced by C2-ceramide by up-regulating the EGFR/JNK pathway. a) 1×10^4^ G3-transfected and vector-transfected MT-1, MDA-MB-468, 66c14, 4T07, and 4T1 cells were inoculated and cultured in 10% FBS/DMEM medium in 96 well culture dishes for 12 hours. After cell attachment, cells were treated with 40 µM for 24 hours. WST-1 assays were used to analyze cell viability. b) G3-transfected and vector-transfected 66c14 cells (1×10^4^) were inoculated and cultured in 10% FBS/DMEM medium in 96 well culture dishes for 12 hours. After cell attachment, we added C2-ceramide (40 µM), and EGF (20 ng/ml), AG 1478 (2.0 µM), PD 98059 (50 µM), or SP 600125 (100 nM) cultured for 24 hours. WST-1 Cell Survival Assays were performed. Compared with vector control group, *n* = 8, * *p*<0.05, **p<0.01, analyzed with t-test. c) Treated with C2-ceramide (40 µM) and EGF (20 ng/ml), AG 1478 (2.0 µM), PD 98059 (50 µM), or SP 600125 (100 nM) for 6 hours, cells were harvested and subjected to immunoblotting with antibodies to pSAPK/JNK, SAPK/JNK, ERK2, pERK, Caspase-3, and β-actin.

66c14 cells expressing versican G3 demonstrated lower cell viability compared with vector control groups when cultured in C2-ceramide (Fig. 3a, 3c, 3d). Annexin V assays confirmed that cell death occurred through apoptosis (Fig. 3b). C2-ceramide is a synthetic lipid, a potent apoptosis inducing substance that has been described as a second messenger of TNF and other stimuli. Immunoblotting showed that the G3 construct enhanced tumor cell apoptosis induced by C2-ceramide through expressing high levels of pSAPK/JNK and caspase-3 (Fig. 3e). During this procedure, G3-transfected cells expressed high level of pERK (Fig. 3e). Lower cell viability was also recorded in G3-expressing MT-1, MDA-MB-468, 4T07, and 4T1 cells after treatment with C2-ceramide (no significance in 4T1 cell line, Fig. 4a). To investigate whether versican G3 promotes cell apoptosis through the EGFR/JNK pathway, we cultured the G3- and vector-transfected 66c14 cells with C2-ceramide, EGF, AG 1478, PD 98059, or SP 600125. We found that versican G3 enhanced cell apoptosis induced by C2-ceramide, an observation inhibited by EGFR inhibitor AG 1478 and SAPK/JNK inhibitor SP 600125 (Fig. 4b). During treatment with C2-ceramide, G3-transfected cells expressed increased pSAPK/JNK and caspase-3, which were also induced by EGF, findings blocked by AG 1478 and SP 600125 but not by PD 98059 (Fig. 4c). SP 600125 also enhanced G3 transfected cells expression of GSK-3β (S9P) when treated with C2-ceramide (Fig. 4c).

### Versican G3 modulated effects on breast cancer cell apoptosis induced by chemotherapeutic agents through the activation of EGFR related signaling

In order to investigate the effects of versican G3 domain on breast cancer cell apoptosis induced by chemotherapeutic drugs, we chose 5 frequently used compounds. Docetaxel is a clinically well established anti-mitotic chemotherapy medication used mainly for the treatment of breast, ovarian, and non-small cell lung cancer [40], [41]. Doxorubicin and Epirubicin are anthracycline antibiotics and work through intercalating DNA strands that result in complex formation that inhibits DNA and RNA synthesis. They also trigger DNA cleavage by topoisomerase II, resulting in mechanisms that lead to cell death. Both agents are commonly used in the treatment of a wide range of cancers [42]. Cyclophosphamide, a nitrogen mustard alkylating agent, from the oxazophorines group [43] was also evaluated. Finally, Trastuzumab is a humanized monoclonal antibody that acts on the HER2/neu receptor and is used principally as an anti-cancer therapy in breast cancer patients whose tumors overexpress this receptor [44].

**Figure 5:**
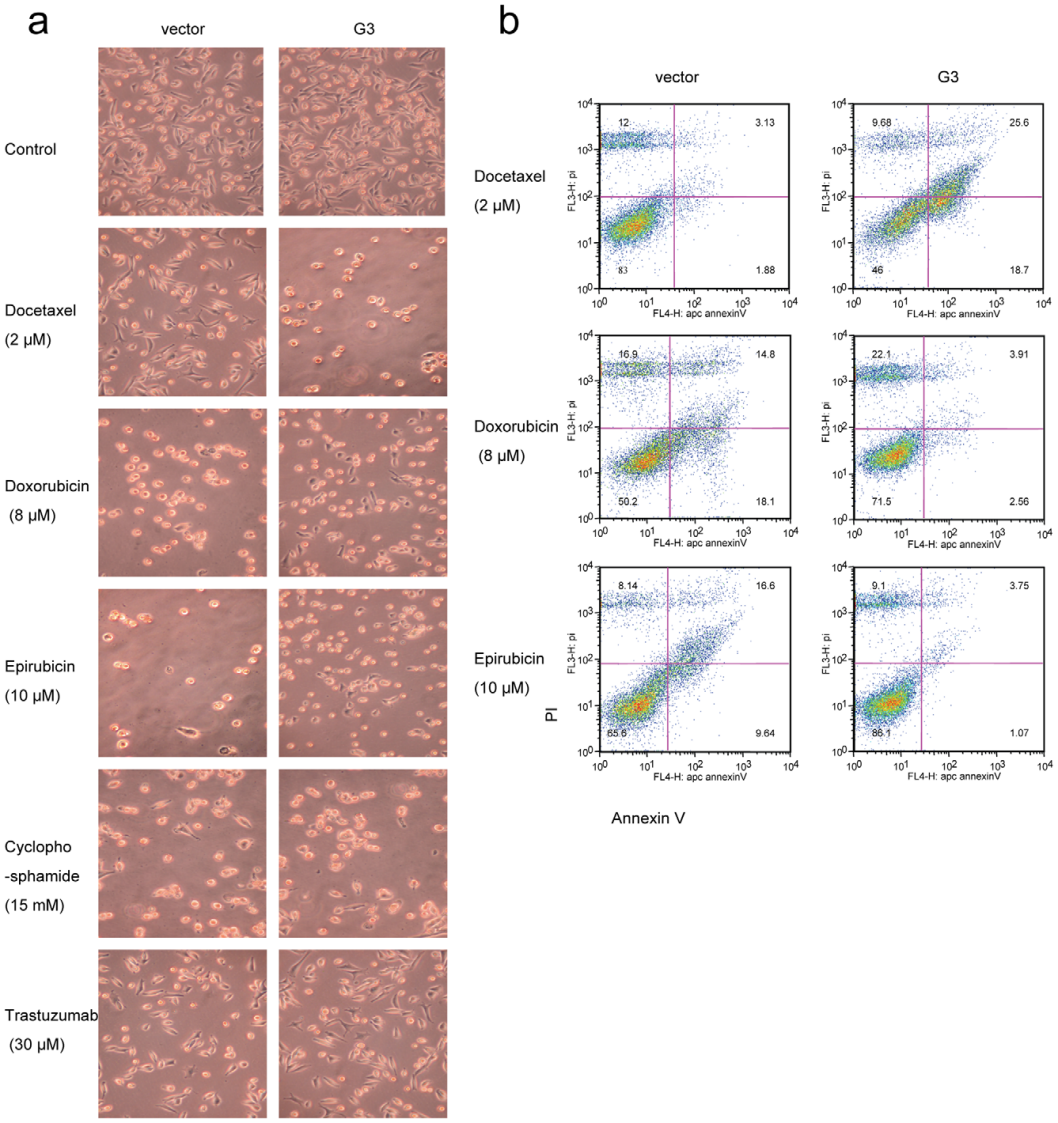
Versican G3 domain enhanced breast cancer cell apoptosis induced by Docetaxel, while cell apoptosis was reduced when treated with Doxorubicin, or Epirubicin. a) G3-transfected and vector-transfected 4T07 cells (1×10^5^) were inoculated in 12 well culture dishes. After cultured for 12 hours, all samples were treated with 2 µM Docetaxel, 8 µM Doxorubicin, 10 µM Epirubicin, 15 mM Cyclophosphamide, or 30 µM Trastuzumab for 24 hours. Cell viability was observed by light microscopy. b) After treated with 2 µM Docetaxel, 8 µM Doxorubicin, or 10 µM Epirubicin for 2 hours, all the samples were subjected to Annexin V assays.

**Figure 6:**
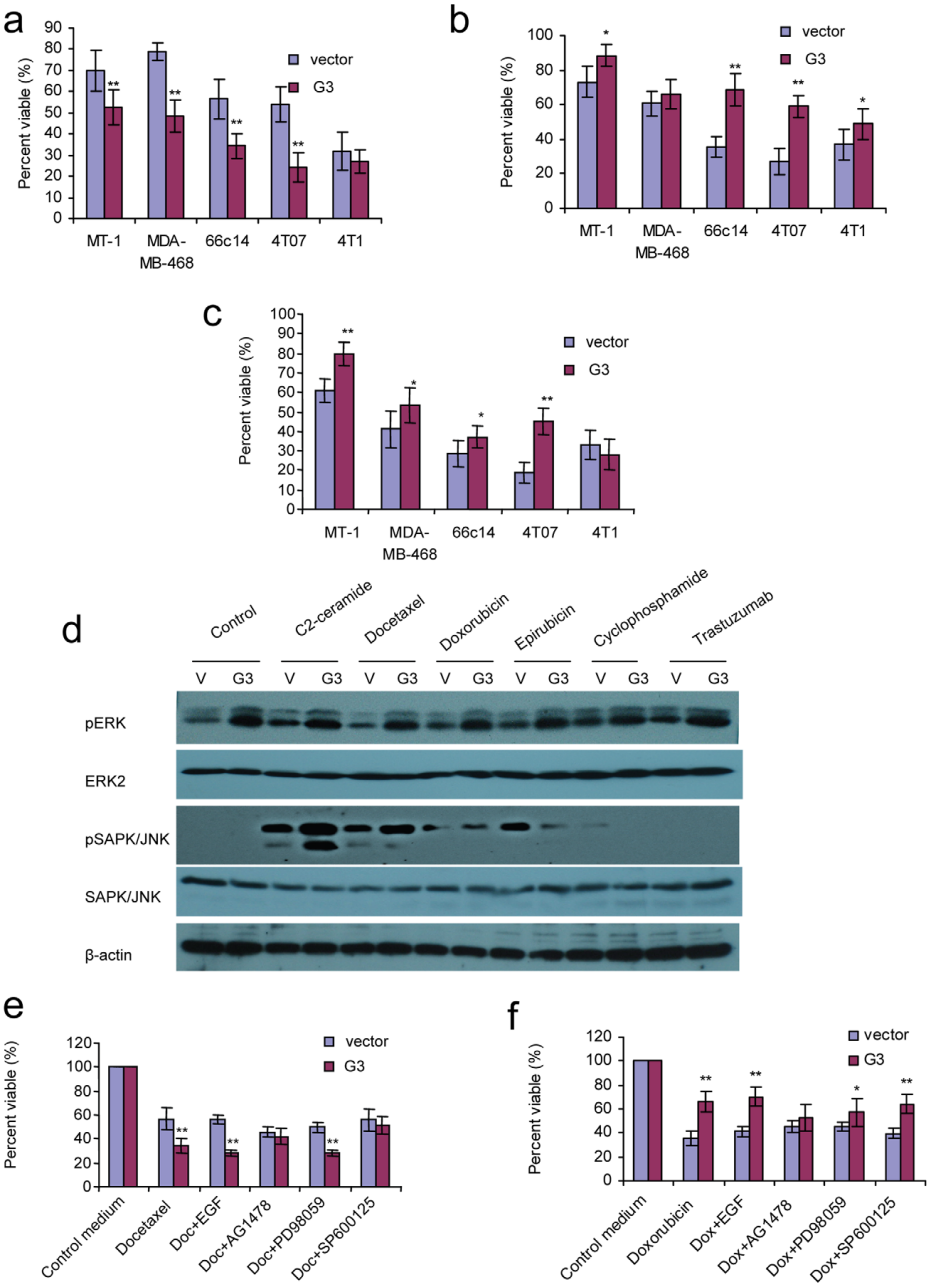
Versican G3 modulated breast cancer cell apoptosis induced by chemotherapeutic drugs through activation of EGFR related signaling. a) 1×10^4^ G3- and vector-transfected MT-1, MDA-MB-468, 66c14, 4T07, and 4T1 cells were inoculated and cultured in 10% FBS/DMEM medium in 96 well culture dishes for 12 hours. After cell attachment, cells were treated with 2 µM Docetaxel for 24 hours. Cell viability was tested by WST-1 assays. b) All cells were treated with 8 µM Doxorubicin, and subjected to WST-1 assays. c) All cells were treated with 10 µM Epirubicin, and subjected to WST-1 assays. Compared with vector control group, *n* = 6, * *p*<0.05, **p<0.01, analyzed with *t*-test. d) Treated with 40 µM C2-ceramide, 2 µM Docetaxel, 8 µM Doxorubicin, 10 µM Epirubicin, 15 mM Cyclophosphamide, or 30 µM Trastuzumab for 6 hours, G3-expressing and vector- expressing 66c14 cells were processed to lysates and subjected to immunoblotting with antibodies to pSAPK/JNK, SAPK/JNK, ERK2, pERK, and β-actin. e) G3-transfected and vector-transfected 66c14 cells (1×10^4^) were inoculated and cultured in 10% FBS/DMEM medium in 96 well culture dishes for 12 hours. After cell attachment, we added Docetaxel (2 µM), and EGF (20 ng/ml), AG 1478 (2.0 µM), PD 98059 (50 µM), or SP 600125 (100 nM) cultured for 24 hours. WST-1 Cell Survival Assays were used to analyze cell viability. f) G3-transfected and vector-transfected 66c14 cells (1×10^4^) were also treated with Doxorubicin (8 µM), and EGF (20 ng/ml), AG 1478 (2.0 µM), PD 98059 (50 µM), or SP 600125 (100 nM) cultured for 24 hours. Cell viability was analyzed by WST-1 assays. Compared with vector control group, *n* = 8, * *p*<0.05, **p<0.01, analyzed with *t*-test.

Analysis by light microscopy revealed that G3-transfected 4T07 cells showed increased cell apoptosis induced by Docetaxel, however, there was a reduction in cell apoptosis when treated with Doxorubicin, or Epirubicin. There was no appreciable difference between G3-transfected cells and the vector- cells after they were treated with Cyclophosphamide or Trastuzumab (Fig. 5a). Annexin V apoptosis assays confirmed that apoptosis was enhanced in G3 expressing cells when treated with Docetaxel, while apoptosis decreased when cultured with Doxorubicin and Epirubicin. WST-1 assays showed that versican G3- transfected MT-1, MDA-MB-468, 66c14, 4T07 cells expressed lower viability when treated with Docetaxel while higher viability was observed when cells were cultured in Doxorubicin and Epirubicin (Fig. 6a, 6b, 6c). However there is no significance for 4T1 cells when treated with Docetaxel, and also no significance for MDA-MB-468 when treated with Doxorubicin. The expression of endogenous versican probably makes the effect of function of exogenously expression of versican G3 not so obviously. Higher expression of versican in 4T1 cell line than other three mouse breast cancer cell lines supports above explanation [20]. MDA-MB-468, a human breast cancer cell line with a very high number of EGF receptors [45], shows less EGFR enhanced when trasfected with versican G3 domain. This may be the main reason why the G3 expressing MDA-MB-468 shows less chemical sensitivity to chemicals. Immunoblotting showed that G3-expressing cells increased p-ERK expression in the chemically treated and non-treated samples. When treated with C2-ceramide or Docetaxel, G3-expressing cells expressed a dramatically high level of pSAPK/JNK, while Doxorubicin and Epirubicin did not significantly impact expression of pSAPK/JNK in G3-expressing cells (Fig. 6d). WST-1 Cell Survival Assays showed that versican G3 enhanced cell apoptosis induced by Docetaxel, an observation blocked by AG 1478 and SP 6000125 (Fig. 6e); it was also observed that cell apoptosis decreased in the presence of Doxorubicin, a finding blocked by AG 1478 and PD 98059 (Fig. 6f).

### Reduction of endogenous versican expression by siRNA (or by linking G3 domain with 3′UTR) prevented G3 modulated effects on cell apoptosis induced by chemotherapeutic drugs

**Figure 7:**
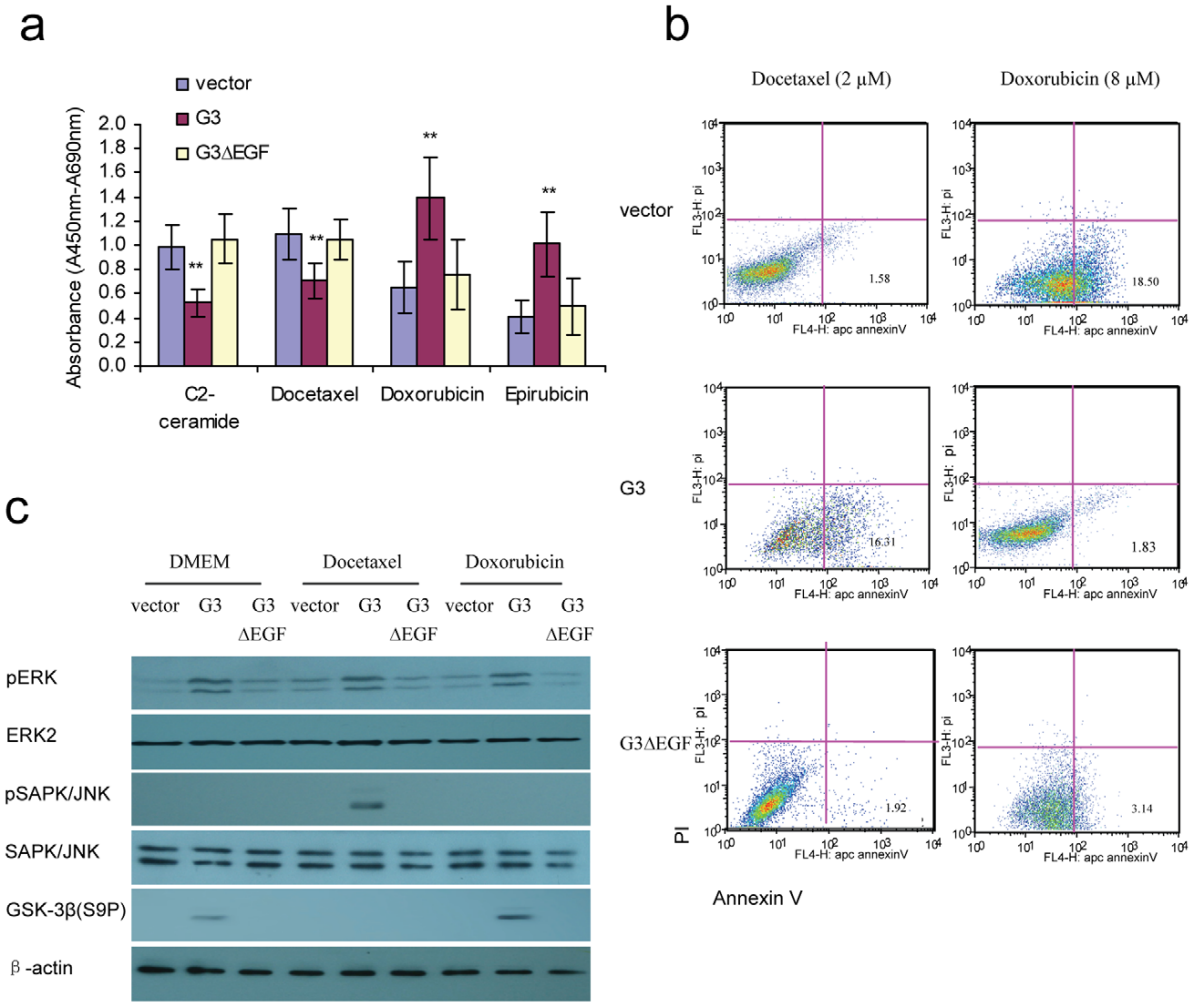
The roles of epidermal growth factor-like motifs of versican G3 domain in modulating breast cancer cell apoptosis induced by chemotherapeutic drugs. a)1×10^4^ G3-, G3ΔEGF- and vector-transfected 4T07 were inoculated and cultured in 10% FBS/DMEM medium in 96 well culture dishes for 12 hours. After cell attachment, cells were treated with 40 µM C2-ceramide, 2 µM Docetaxel, 8 µM Doxorubicin, or 10 µM Epirubicin for 24 hours. Cell viability was tested by WST-1 assays. b) After treated with 2 µM Docetaxel or 8 µM Doxorubicin for 1 hour, samples were subjected to Annexin V assays. c) Treated with 2 µM Docetaxel or 8 µM Doxorubicin all cells were processed to lysates and subjected to immunoblotting with antibodies to pSAPK/JNK, SAPK/JNK, ERK2, pERK, GSK-3β (S9P), and β-actin.

The key functions of the EGF-like motifs of versican G3 domain were well demonstrated by our former study [31], [32], [46]. Here we found that G3 fragment lacking the EGF-like motifs (G3ΔEGF) construct transfected 4T07 cells did not show enhanced cell apoptosis when treated with C2-ceramide or Docetaxel, and also did not show enhanced anti-apoptosis when cultured in Doxorubicin or Epirubicin as G3 transfected cells (Fig. 7a, 7b). Immunoblotting indicated that G3ΔEGF expressing cells did not showed enhanced pERK as G3 expressing cells. G3ΔEGF expressing cells also did not showed enhanced pJNK when treated with Docetaxel and enhanced GSK-3β(S9P) when cultured in Doxorubicin as G3 expressing cells.

Immunoblotting and RT-PCR showed that versican V1 isoform expressed differently in the four human breast cell lines. It was expressed highly in MT-1, MDA-MB231 and MDA-MB-468 cells, and low levels were observed in MCF-7 cells (Fig S1a). The anti-versican siRNA that has been confirmed to be able to silence vesicant expression [47] was used to transfect MT-1 cells, and it revealed significant versican V1 mRNA and protein down-regulation through RT-PCR and immunoblotting (Fig S1b). The western blot results presented here are obtained using the antibody from abcam (ab19345) which is indicated suitable for detection of versican V1 isoform, and shows only one band -versican V1, 250–300 kDa. We then examined the expression of pERK, ERK, pSAPK/JNK, SAPK/JNK in anti-versican siRNA expressing MT-1 cells treated with Docetaxel, Doxorubicin, or Epirubicin. Immunoblotting showed that the expression of pERK V1 was down-regulated in the anti-versican siRNA expressing MT-1 cell, irrespective of whether or not it was chemically treated, and there was no significant change in the expression of pSAPK/JNK (Fig S1c). WST-1 assays showed that versican G3 promoted cell apoptosis induced by C2-ceramide and Docetaxel, whereas cell apoptosis induced by Doxorubicin and Epirubicin was reduced. While the anti-versican siRNA- transfected cells showed a reduction in the extent of cell apoptosis induced by C2-ceramide, we observed enhanced effects on cell apoptosis induced by Doxorubicin and Epirubicin when compared with G3-transfected and vector-transfected cells (Fig S1d).

In order to further confirm the role of G3 in apoptosis, we linked the G3 domain with versican 3′-UTR (Fig S2a). Our previous research indicated that G3-3′ UTR transfected cells expressed lower G3 protein compared to G3 expressing cells [48]. So we can use the G-UTR construct to observe the effect of decreasing expression of G3 in G3 expressing cells. Immunoblotting demonstrated that G3-3′ UTR stably transfected 66c14 cells expressed much lower levels of G3 protein than the G3 transfected cells (Fig S2b). The microscopic morphology of G3 transfected cells was quite different from the vector control cells. The G3 expressing cells spread evenly on the culture dishes, while the vector control cells were prone to cell aggregation. The G3-3′ UTR- expressing cells appeared between these two different morphologies. G3-3′ UTR- transfected cells neither promoted the extent of cell apoptosis induced by C2-ceramide or Docetaxel, nor enhanced cell survival when treated with Doxorubicin or Epirubicin (Fig S2c, S2d). Our experiments demonstrate that the sensitivity of breast cancer cells to chemotherapeutically induced apoptosis was versican G3 domain dependant.

## Discussion

Increased activation of EGFR and dysregulated expression of versican contributes towards a more aggressive human breast cancer phenotype [15], [16], [49], [50], [51]. Targeted therapies shows considerable promise for the future of cancer treatment and much attention has been focused on developing inhibitors of the EGFR-mediated signaling pathway [49], [52], [53]. Evidence that EGFR signaling promotes cell proliferation, cell survival and metastasis supports current efforts to identify approaches that inhibit this pathway [54], [55], [56]. Anti-EGFR immunotherapeutics in cancer treatment is undergoing intensive study [57]. The efficacy of Erlotinib and Gefitinib in treating breast cancer is currently being tested in various phases of clinical trials either as single agent treatment or in combination with other agents such as Docetaxel, Gemcitabine, Paclitaxel [58], [59], [60]. The overall efficacy of anti-EGFR treatments to date remains moderate and there is desire to enhance results that will occur through a better mechanistic understanding of the signaling pathway [61]. A phase II study of using Erlotinib and Gemcitabine demonstrated lower than anticipated effects on patients with metastatic breast cancer [58] while a Phase I study applying Gefitinib and Docetaxel demonstrated encouraging anti-tumor activity as a first-line chemotherapy in metastatic breast cancer [60].

Abnormal expression of proteoglycans (PGs), such as versican, in cancer and stromal cells may serve as a biomarker for tumor progression and patient survival [62]. Enhanced understanding of the regulation and involvement of versican in cancer may offer a novel approach to cancer therapy by targeting the tumor microenvironment [62]. The effect of signaling pathways on versican synthesis can be reversed following treatment with various tyrosine kinase inhibitors [63]. The tyrosine kinase inhibitor genistein can block versican expression induced by growth factors in malignant mesothelioma cell lines [64]. Therefore, targeting versican synthesis may be a potential mechanism for reducing this powerful tumor-promoting agent. Genetic and preclinical studies support the targeting of growth factor signaling as a therapeutic strategy for combating cancer. Individuals with overexpression of versican in breast cancer may more likely benefit from anti-EGFR therapy given known effects of EGF-like motifs in versican, a scientific consideration that warrants further evaluation. However, there are no data to show that such approaches are effective in inhibiting the effects of versican in cancer cell models.

The presence of two EGF-like domains in versican G3 and the importance of versican as a prognostic factor in breast cancer motivates further research in delineating the role of EGF receptors and the downstream signaling pathways in invasive breast cancer [19]. Versican G3 domain appears to be important in local and systemic invasiveness of human breast cancer [19]; our previous investigation demonstrated that versican G3 domain enhanced breast cancer cell growth, migration and systemic metastasis by up-regulating the EGFR-mediated signaling pathway [20]. Both selective EGFR inhibitor AG 1478 and selective MEK inhibitor PD 98059 were observed to be able to block this signaling pathway and prevent versican G3 induced effects on mammary cancer cell proliferation. In the present study, we have focused on the role of versican G3 domain in modulating breast cancer cell apoptosis. Breast cancer cell apoptosis appears to be a factor associated with cancer cell sensitivity or resistance to chemotherapy and mechanisms appear influenced by EGFR signaling. The particular activation or inhibition of downstream EGFR signaling appears to influence cancer cell apoptotic responses to versican mediated effects and appear variably modulated dependant on chemotherapeutic drug or EGFR inhibitor delivered.

It has been reported that versican and its G3 domain possess properties that promote cell growth and survival in low serum and serum-free conditions in breast cancer cells [19], [20]. Versican has also been described to contribute an important role in reducing oxidant injury through an enhancement of cell-matrix interactions [8]. Integrin-β1 was reported to reduce radical-induced apoptosis by binding to G3 domain [65]. In the current study, we demonstrated that versican G3-expressing breast cancer cells express enhanced cell survival in serum free medium and in response to certain chemotherapeutic drugs such as Doxorubicin and Epirubicin. G3 expressing cells demonstrated a greater viability in serum free medium and chemotherapeutic drugs such as Doxorubicin or Epirubicin, which expressed activated EGFR/ERK signaling. pERK, GSK-3β (S9P) and CDK2 levels were continually recorded at high levels in G3-expressing cells. Recent advances in the mechanisms of oncogenesis have revealed that the constitutive activation of the EGFR/ERK pathway allows the tumor cells to bypass regulatory check points that normally balance cell growth and cell apoptosis thereby activating cell cycle entry. Effective chemotherapy may induce cellular damage on a massive scale because it can engage one or more of these check points or drive cancer cells towards apoptosis [66]. Activation of CDK2 and pERK, and that the bypass of regulatory controls in cell cycle progression and cell apoptosis appear to significantly influence tumor growth and survival [66]. Activated glycogen synthase kinase-3ß serine-9 phosphorylation (GSK-3β, S9P) is also required for tumor cell survival and anti-apoptosis [67]. Based on that the present study, enhanced expression of pERK, GSK-3β (S9P) and CDK2 in G3- expressing breast cancer cells favored cell survival and growth even in serum free conditions or when cultured in the environment of applied chemotherapeutic reagents. In particular, versican G3 enhanced cell survival was prevented by both selective EGFR inhibitor AG 1478 and selective MEK (ERK kinase) inhibitor PD 98059 through mechanisms blocking G3 activated expression of pERK and GSK-3 β (S9P). Versican G3-expressing breast cancer cells demonstrated enhanced cell survival in serum free medium and chemotherapy by activating EGFR/ERK signaling and its down-stream pathway proteins CDK2 and GSK-3β (S9P). To validate the roles of versican and G3 domain in modulating breast cancer cell apoptosis in response to applied chemotherapy, we transfected tumor cells with anti-versican siRNA as well as by linking versican G3 domain with versican 3′-UTR (G3-UTR) that reduces versican and G3's functionality. Prior study demonstrated that non-coding versican 3′-UTR significantly down-regulates G3 protein expression [48]. Concordantly, we observed that both anti-versican siRNA and G3-UTR construct reduced G3 enhanced anti-apoptosis when treated with Doxorubicin and Epirubicin.

The EGFR signaling pathway is indispensable for cell cycle progression while it may also efficiently enhance apoptosis [68]. Although activation of the EGFR/ERK signaling pathway is generally considered to lead to cell survival [69], there is evidence that in certain conditions it may also transmit pro-apoptotic signals [69], [70]. In addition to its effects on proliferative capacity and increasing apoptotic resistance, over-expression of versican can be accompanied by selective sensitization to apoptosis [7]. Whereas V1-transfected cells have shown resistance to apoptosis, they also have become significantly sensitized to other apoptotic stimuli, including UV radiation, chemotherapeutics, hypoxic mimetics, and conjugated linoleic acid. Elevated resting levels of the tumor suppressor p53 play a key role in inducing apoptosis in response to various detrimental events, including DNA damage, hypoxia, and telomere erosion [7]. In this study we also noted that versican G3 expressing breast cancer cells showed enhanced apoptosis when treated with certain chemicals, such as C2-ceramide and Docetaxel. In this scenario, chemotherapy induced apoptosis may be enhanced due to the recruitment of enhanced efficiency of cellular signaling. We found that although high levels of pERK were observed in G3-expressing cells when treated with these chemicals, one of the other EGFR down-stream proteins p-SAPK/JNK was dramatically activated. The pro-death or pro-survival role of ERK can have both, survival or cell death activities [22], [71]. Literature supports an effect of breast cancer cells on cellular SAPK/JNK activation in a pro-death capacity but a role of pro-survival was also observed [72]. In our study, both p-ERK and p-JNK was expressed in high levels in the G3-expressing cells after treatment with C2-ceramide and Docetaxel. To determine which factor played a key role in versican G3 enhanced cell apoptosis, we co-treated the G3-expressing cells with chemicals and AG 1478, PD 98059 or SP 600125; we observed that G3 enhanced effects on cell apoptosis was blocked by AG 1478 and SP 600125 but was not appreciably by PD 98059. This supports versican G3 promotion of tumor cell apoptosis induced by C2-ceramide and Docetaxel occurring through EGFR/JNK mediated signaling. Persistently high levels of p-SAPK/JNK observed in G3-expressing breast cancer cells resulted in an increase of one of the key mediators of mammalian cell apoptosis (i.e. Caspase-3), which consequently led to cell death. This hypothesis was supported by the fact that both AG 1478 and SP 600125 blocked G3 enhanced expression of Caspase-3 and cell apoptosis while PD 98059 did not. Reduction in expression of versican and versican G3 domain by anti-versican siRNA and G3-3′UTR construct significantly reduced G3 enhanced effects on cell apoptosis induced by chemotherapeutics and confirmed that versican G3-expressing breast cancer cells promoted cell apoptosis induced by chemotherapeutics through G3 dependant mechanisms.

**Figure 8:**
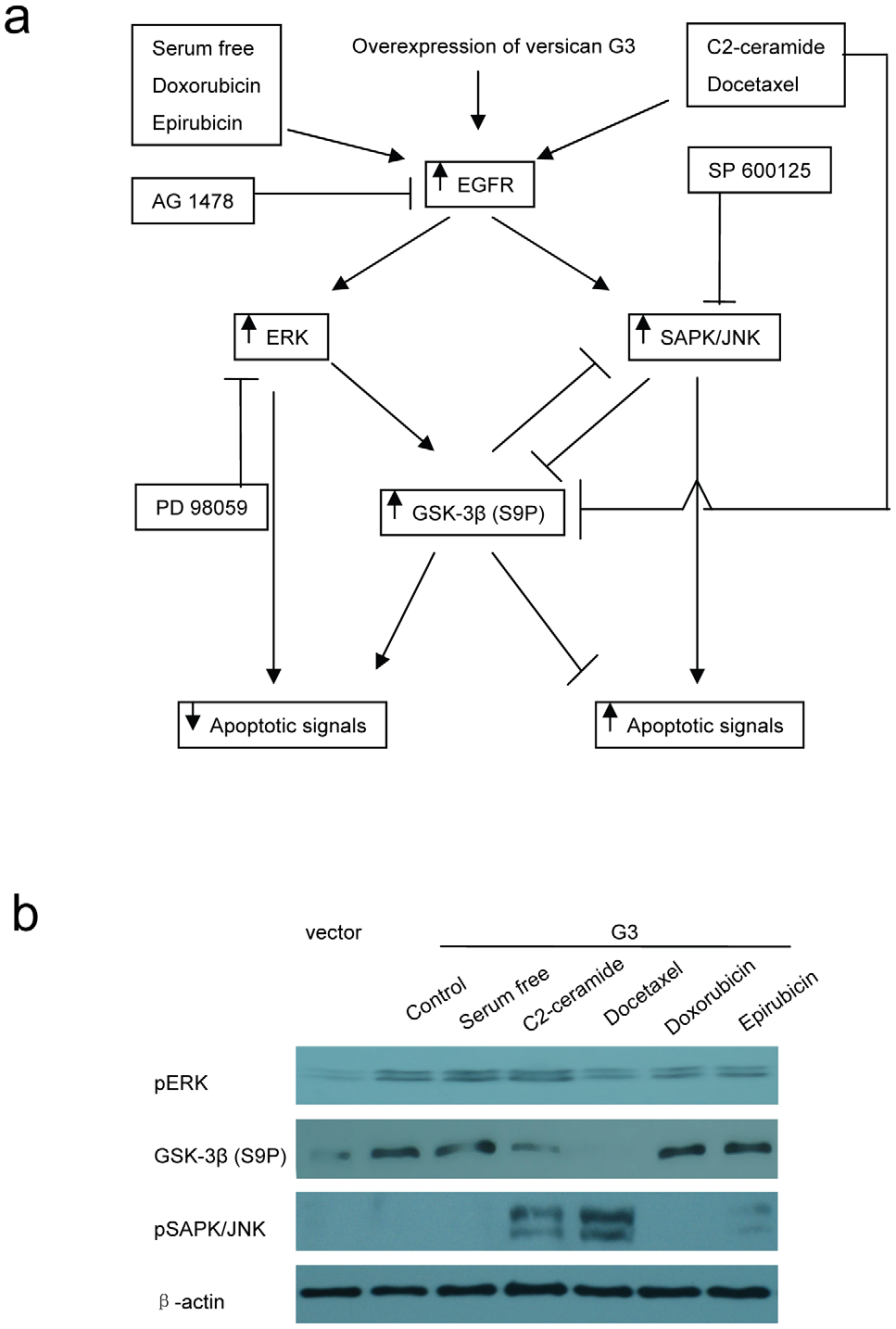
Versican G3 domain modulates breast cancer cell apoptosis in response to chemotherapy and EGFR targeting therapy. a) A model of versican G3 in modulating breast cancer cell apoptosis in chemotherapy and EGFR targeting therapy. The differential responses to chemical induced apoptosis depend on activation and balance of EGFR signaling and its further enhanced down stream pathway. Overexpressing of versican G3 in breast cancer cell enhances expression of EGFR signal and its downpathways. Usually it will process to ERK and GSK pathway, which expresses enhanced cell survival. The GSK works as a key point, which inhibits expression of JNK and makes the cell process to the ERK pathway. Expression of JNK may induce cell apoptosis. Some chemicals like c2-ceramide and Docetaxel can directly inhibit expression of GSK, which alleviates its inhibition on JNK pathway. Expression of JNK can also inhibit expression of GSK, making the G3 expression cells process to apoptosis. b) Vector and G3 transfected 66c14 cells (1×10^6^) were inoculated and cultured in 10% FBS/DMEM medium in 6 well culture dishes for 12 hours. After cell attachment, cells were treated with serum free DMEM medium for 4 days or treated with 40 µM C2-ceramide, 2 µM Docetaxel, 8 µM Doxorubicin, or 10 µM Epirubicin for 2 hours. Cell lysates prepared were subjected to immunoblotting with antibodies to pERK, GSK-3β (S9P), pSAPK/JNK and β-actin.

An interesting observation of our study is the apparent dual roles of versican G3 domain in modulating breast cancer cell resistance to chemotherapy and EGFR targeting therapy. EGFR signaling appears crucial to the sensitivity or resistance of versican expressing breast cancer cells to chemotherapy. The apoptotic effects of chemotherapeutics on these cells depend on the activation and balance of EGFR signaling and its effects down-stream. Certain chemicals such as Doxorubicin and Epirubicin activate versican G3-expressing cells' endogenous EGFR/ERK/GSK-3β (S9P) signaling promoting chemical resistance while others chemicals appear to enhance these cells' sensitivity to chemotherapy through increased expression of EGFR/JNK signaling and subsequent effects on apoptosis. Our study has identified a key EGFR down stream proteins, GSK-3β (S9P) that appears critically important as a regulatory check-point in the balance of apoptosis and anti-apoptosis (Fig. 8a). Results demonstrated that G3-expressing cells enhanced GSK-3β (S9P) expression when treated with a serum free medium, Doxorubicin or Epirubicin; they also expressed decreased GSK-3β (S9P) and activated pSAPK/JNK when treated with C2-ceramide or Docetaxel. The pERK expression remained at high levels when these cells were treated with different chemicals (Fig. 8b). The increased expression of GSK-3β (S9P) inhibits the expression of pSAPK/JNK, enhancing G3-cell survival. Chemicals such as C2-ceramide and Docetaxel reduce G3-cells expression of GSK-3β (S9P), which alleviates inhibition of pSAPK/JNK activity encouraging the survival system favor cell apoptosis. On the other hand, expression of pSAPK/JNK may also inhibit expression of GSK-3β (S9P), and enhance cell apoptosis (Fig. 8a). Selective JNK inhibitor SP 600125 enhanced G3 cells expression of GSK-3β (S9P) when treated with serum free or C2-ceramide medium suggesting that expression of pSAPK/JNK inhibits expression of GSK-3β (S9P), a pathway leading to cell apoptosis (Fig. 2c, Fig. 4c). A model based on this study of versican G3 modulating breast cancer cell apoptosis in response to chemotherapy and EGFR targeting therapy is shown in Fig. 8a.

Although a large number of new agents targeting the EGFR pathways are being tested and have shown certain efficacy through greater survival in clinical and pre-clinical models, it remains unclear as to how combination EGFR therapy with chemotherapy will impact breast cancer patients. Literature is varied with some clinical trials demonstrating that EGFR targeting agents synergize with cytotoxic chemotherapies [59], [60], while others have failed to show any survival advantage of combination over single-agent therapy in advanced breast cancer patients [58]. These varied effects could potentially be explained by the interaction of EGFR targeting and chemotherapeutics on EGFR signaling and effects of cell cycle entry as well as apoptosis. We have identified that key downstream pathway EGFR signaling proteins such as GSK-3β (S9P) may appear to play a role in how cells respond to treatment. Ongoing study on the mechanisms of cancer invasiveness and cellular signaling will further advance our knowledge on how extracellular matrix and cellular factors such as versican and EGFR signaling impact patient outcomes and can be modulated in response to treatment.

Our study has clinical relevance and motivates additional pre-clinical study towards the development of new clinical agents that can be tested in the treatment of breast cancer. Our mechanistic study on EGFR related signaling demonstrates that chemotherapeutic drugs can have varying effects on signaling that may either positively or negatively impact cancer cell survival through mechanisms that influence apoptosis. Although there are several clinical agents that broadly target EGFR, downstream effects appear to critically influence cellular apoptosis and the development of more specific drugs that can modulate downstream targets such as GSK-3b (S9P) expression as demonstrated by this study is desirable. The field of breast cancer chemotherapeutics is also evolving with recent interest in neoadjuvant approaches to treatment which serves as a valuable research platform to test patient specific primary tumor response to systemic therapies prior to surgery in early disease thereby helping to refine patient selection for therapy limiting treatment specifically to those that are most likely to benefit from systemic agents many of which possess significant toxicity profiles.

## Supporting Information

Figure S1 **Silencing versican expression using siRNA.** a) MT-1, MDA-MB-231, MCF-7, MDA-MB-468 cell lysates were subjected to immunoblotting and RT-PCR. b) MT-1 cells were stably transfected with anti-versican siRNA. Versican V1 expression was analyzed by immunoblot and RT-PCR. c) The expression of pERK, ERK, pSAPK/JNK, SAPK/JNK of vector-expressing and anti-versican siRNA- expressing MT-1 cells was analyzed by immunoblotting, after treatment with 2 µM Docetaxel, 8 µM Doxorubicin, or 8 µM Epirubicin for 6 hours. d) WST-1 assays were used to test cell viability of vector-, versican G3-transfected, and anti-versican siRNA- transfected MT-1 cells, which were treated with 40 µM C2-ceramide, 2 µM Docetaxel, 8 µM Doxorubicin, or 10 µM Epirubicin for 24 hours.
(TIF)

Figure S2 **Reduction of versican G3's function using versican G3-UTR.** a) Versican G3 domain was linked with or without the 3′UTR of versican, producing G3 and G3-UTR constructs. b) Cell lysates prepared from 66c14 cells stably transfected with versican G3 and G3-UTR construct were subjected to immunoblotting. c) Vector-transfected, G3-UTR-transfected, and G3- transfected 66c14 cells (1×10^5^) were inoculated in 12 well culture dishes. After culture for 12 hours, all samples were treated with 40 µM C2-ceramide, 2 µM Docetaxel, 8 µM Doxorubicin, or 10 µM Epirubicin for 24 hours. Cell viability was analyzed by light microscopy. d) Vector, G3-UTR, and G3 transfected 66c14 cells (1×10^4^) were inoculated and cultured in 10% FBS/DMEM medium in 96 well culture dishes for 12 hours. After cell attachment, cells were treated with 40 µM, C2-ceramide, 2 µM Docetaxel, 8 µM Doxorubicin, or 10 µM Epirubicin for 24 hours. Cell viability was analyzed by WST-1 assays. Compared with vector control group, *n* = 6, * *p*<0.05, **p<0.01, analyzed with *t*-test.
(TIF)
