# Correction: Versican G3 Domain Modulates Breast Cancer Cell Apoptosis: A Mechanism for Breast Cancer Cell Response to Chemotherapy and EGFR Therapy

**DOI:** 10.1371/annotation/fa027b28-3589-4307-8fc0-ff80fb79e440

**Published:** 2012-01-04

**Authors:** William Weidong Du, Burton B. Yang, Bing L. Yang, Zhaoqun Deng, Ling Fang, Sze Wan Shan, Zina Jeyapalan, Yaou Zhang, Arun Seth, Albert J. Yee

There was an error in the funding statement. The correct funding statement is: This work was supported by a grant from CIHR China-Canada Joint Health Research Initiative Grant (CCI 102929) to BBY who is the recipient of a Career Investigator Award (CI5958) from the Heart and Stroke Foundation of Ontario. Support for this study was also provided, in part, from the Holland Musculoskeletal Program, Sunnybrook Health Sciences Centre. The funders had no role in study design, data collection and analysis, decision to publish, or preparation of the manuscript. 

