# Supplementary figures and images for: Versican G3 Domain Modulates Breast Cancer Cell Apoptosis: A Mechanism for Breast Cancer Cell Response to Chemotherapy and EGFR Therapy
Source: PLoS One. 2011 Nov 9;6(11):e26396. doi: 10.1371/journal.pone.0026396 (PMC3212514; doi:10.1371/journal.pone.0026396)

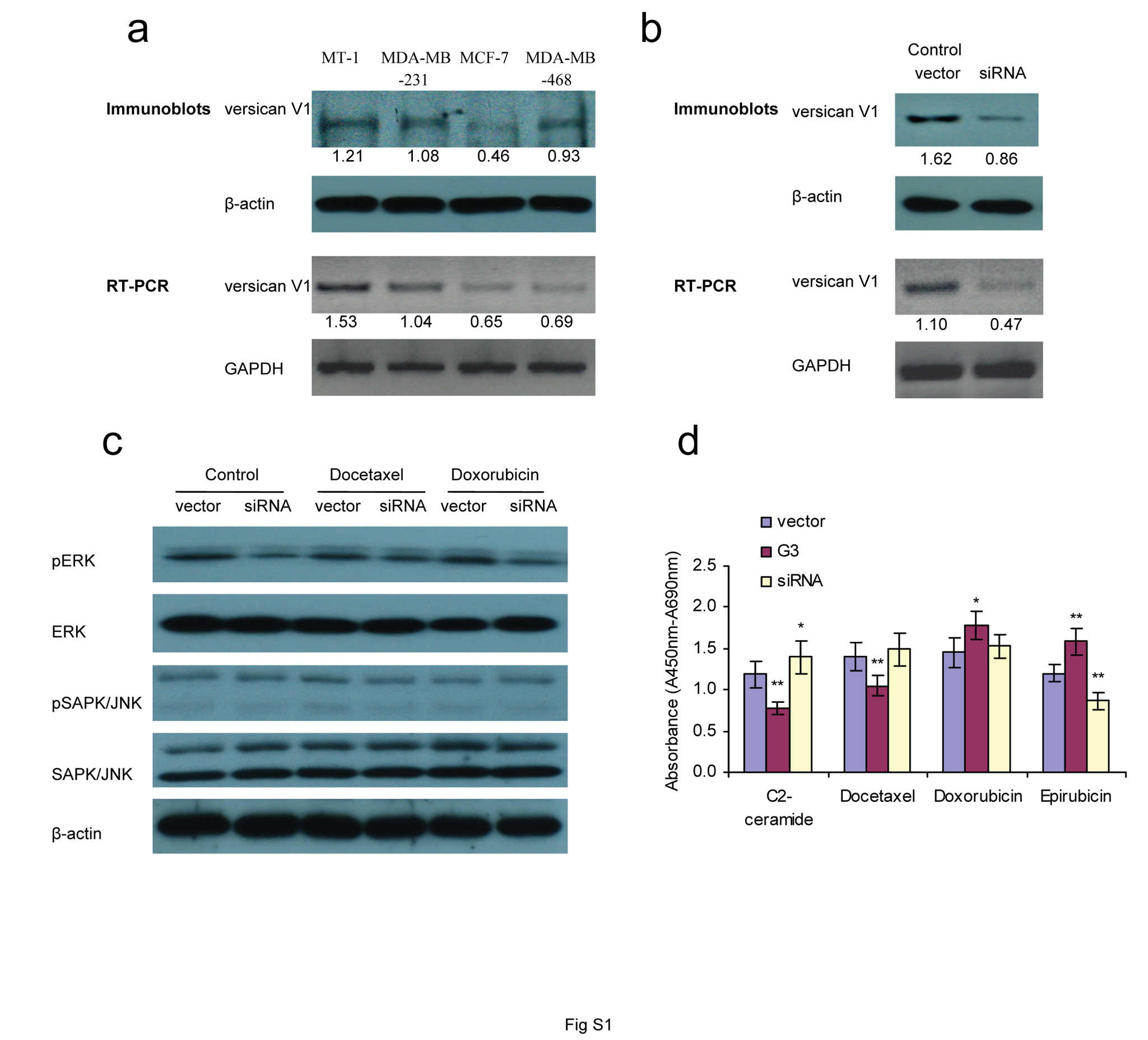

Supplement: Figure S1 — Silencing versican expression using siRNA. a) MT-1, MDA-MB-231, MCF-7, MDA-MB-468 cell lysates were subjected to immunoblotting and RT-PCR. b) MT-1 cells were stably transfected with anti-versican siRNA. Versican V1 expression was analyzed by immunoblot and RT-PCR. c) The expression of pERK, ERK, pSAPK/JNK, SAPK/JNK of vector-expressing and anti-versican siRNA- expressing MT-1 cells was analyzed by immunoblotting, after treatment with 2 µM Docetaxel, 8 µM Doxorubicin, or 8 µM Epirubicin for 6 hours. d) WST-1 assays were used to test cell viability of vector-, versican G3-transfected, and anti-versican siRNA- transfected MT-1 cells, which were treated with 40 µM C2-ceramide, 2 µM Docetaxel, 8 µM Doxorubicin, or 10 µM Epirubicin for 24 hours. (TIF) [file pone.0026396.s001.tif]

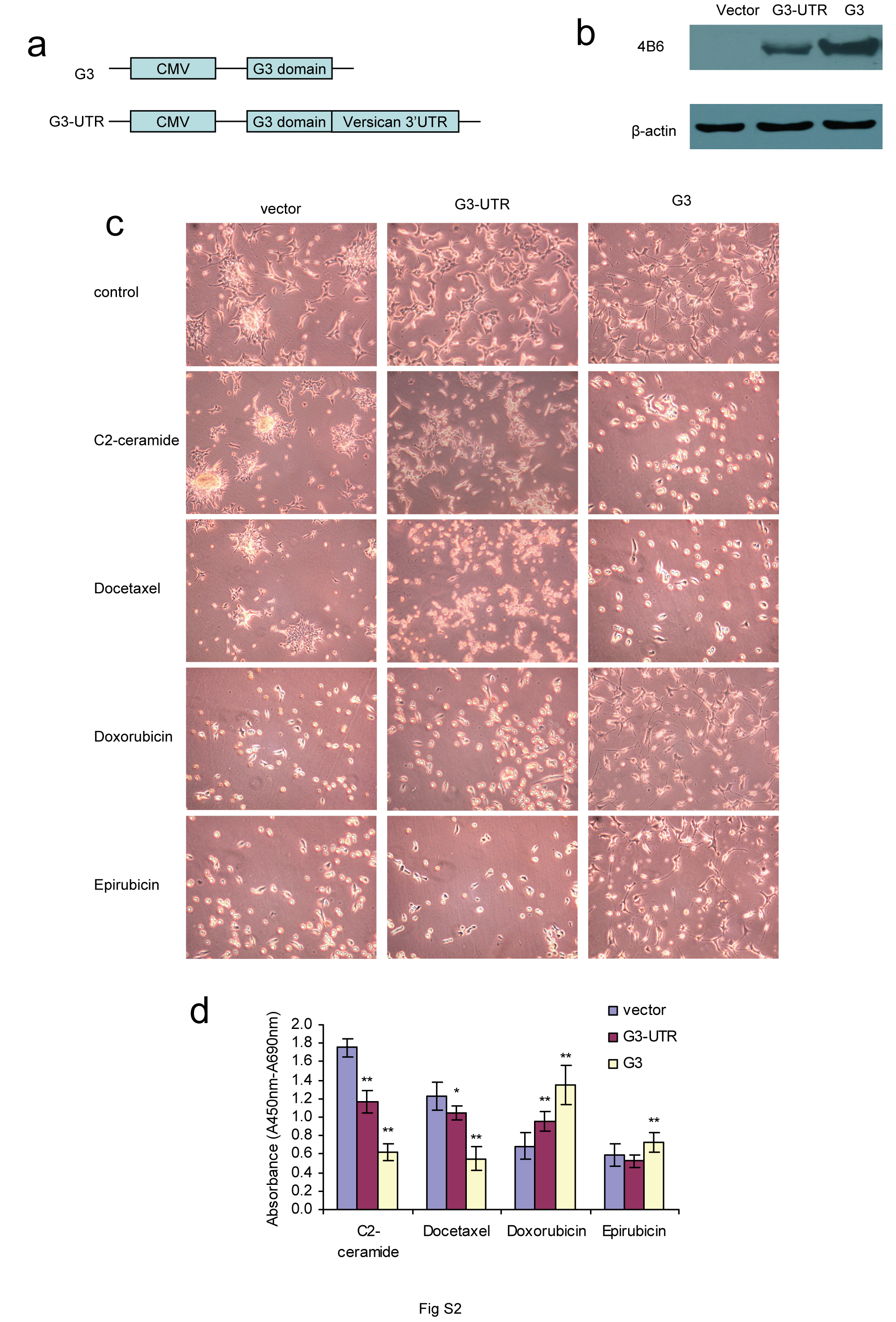

Supplement: Figure S2 — Reduction of versican G3's function using versican G3-UTR. a) Versican G3 domain was linked with or without the 3′UTR of versican, producing G3 and G3-UTR constructs. b) Cell lysates prepared from 66c14 cells stably transfected with versican G3 and G3-UTR construct were subjected to immunoblotting. c) Vector-transfected, G3-UTR-transfected, and G3- transfected 66c14 cells (1×10^5^) were inoculated in 12 well culture dishes. After culture for 12 hours, all samples were treated with 40 µM C2-ceramide, 2 µM Docetaxel, 8 µM Doxorubicin, or 10 µM Epirubicin for 24 hours. Cell viability was analyzed by light microscopy. d) Vector, G3-UTR, and G3 transfected 66c14 cells (1×10^4^) were inoculated and cultured in 10% FBS/DMEM medium in 96 well culture dishes for 12 hours. After cell attachment, cells were treated with 40 µM, C2-ceramide, 2 µM Docetaxel, 8 µM Doxorubicin, or 10 µM Epirubicin for 24 hours. Cell viability was analyzed by WST-1 assays. Compared with vector control group, n = 6, * p<0.05, **p<0.01, analyzed with t-test. (TIF) [file pone.0026396.s002.tif]
